# Sustainable Production of Poly(3-hydroxybutyrate) Using Eucalyptus Bark: Integration with Green Downstream Processing

**DOI:** 10.1021/acssuschemeng.5c14243

**Published:** 2026-02-09

**Authors:** João Matias, Thomas Rodrigues, Cristiana A. V. Torres, Susana Marques, Belina Ribeiro, Francisco Gírio, Maria A. M. Reis, Filomena Freitas

**Affiliations:** † Associate Laboratory i4HB, Institute for Health and Bioeconomy, School of Science and Technology, NOVA University Lisbon, 2829-516 Caparica, Portugal; ‡ UCIBIO—Applied Molecular Biosciences Unit, Department of Chemistry, School of Science and Technology, NOVA University Lisbon, 2829-516 Caparica, Portugal; § Unidade de Bioenergia e Biorrefinarias, Laboratório Nacional de Energia e Geologia I.P., 2610-999 Lisboa, Portugal

**Keywords:** eucalyptus bark, enzymatic hydrolysis, polyhydroxyalkanoates (PHA), *Burkholderia thailandensis*, alcalase

## Abstract

This study integrates the valorization of a lignocellulose material into poly­(3-hydroxybutyrate), P­(3HB), with biopolymer extraction from bacterial cells with the enzyme alcalase. The work focused on *Burkholderia thailandensis* DSM 13276 as the P­(3HB) producer and on eucalyptus bark, a byproduct from the pulp industry, as the sole feedstock for bacterial cultivation. The eucalyptus bark was hydrolyzed by a cellulolytic enzymatic cocktail following steam explosion and further subjected to ultrafiltration for enzyme recovery. The resulting hydrolysate supported good cell growth, achieving a cell dry weight of 7.67 ± 0.16 g/L within 72 h of cultivation, and high P­(3HB) content (60.0 ± 2.19 wt %) in the bacterial cells, clearly favoring biopolymer synthesis over cell growth, as demonstrated by the polymer and growth yields (0.190 g_P(3HB)_/g_sugar_ and 0.026 g_X_/g_sugar_, respectively). High extraction efficiency (96%) and biopolymer purity (100 ± 3.38%) were reached by enzymatic treatment, resulting in a sample with properties aligned with those of commercial P­(3HB) in terms of molecular mass distribution, crystallinity, and thermal properties. These findings demonstrate the successful use of a sustainable feedstock together with the application of environmentally friendly technologies based on the use of enzymes for both lignocellulosic saccharification and biopolymer recovery to develop high-quality bioplastics, advancing the goals of a circular bioeconomy.

## Graphical abstract

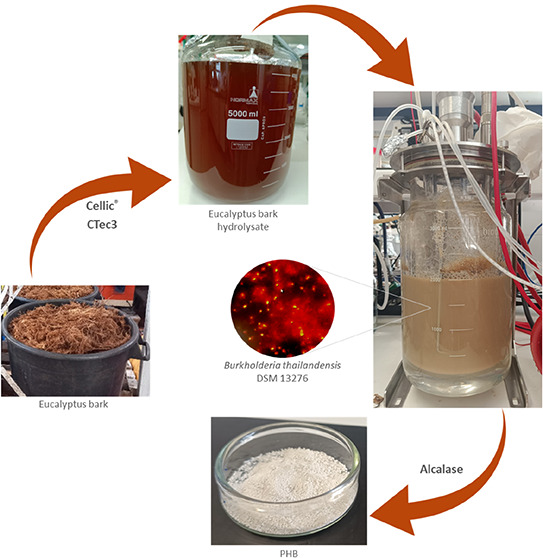

## 1 Introduction

Polyhydroxyalkanoates (PHAs) are biobased plastics synthesized by many bacteria that are being explored for their sustainability and reduced environmental impact. PHAs are fully biodegradable, breaking down in the environment into nontoxic byproducts,^1^ and possess thermal and mechanical properties that render them interesting alternatives to several fossil-based plastics in many of their applications.^2^ PHAs’ properties range from thermoplastics with high mechanical resistance (e.g., the homopolymer polyhydroxybutyrate, P­(3HB)) to flexible and elastic elastomers (e.g., medium-chain length PHAs).^3^ Moreover, they are biocompatible, a feature that renders them of great interest for biomedical uses.^4^

Despite these advantages, widespread commercialization is hindered by high production costs. These costs are driven by the requirement for specific carbon substrates and by complex downstream processing. Since PHAs are accumulated intracellularly, their recovery requires energy-intensive cell disruption and rigorous purification steps, which can account for up to 50% or more of the total production expenses, and often imposes a heavy environmental footprint due to the traditional use of toxic halogenated solvents.^5,6^ This inefficiency contributes to a market price of 3.0 to 8.5 €/kg, compared to 1.0 to 2.0 €/kg for traditional plastics.^5,7^ As a strategy to tackle this problem, different renewable feedstocks, including forest and agricultural residues, can be used as substrates by microorganisms to produce PHAs,^8^ promoting waste valorization within a circular bioeconomy concept.^9,10^

However, this approach makes sense only if the extraction process is as sustainable as the raw materials used. To ensure that the benefits of using residues are not lost during purification, it is crucial to develop recovery methods that are both ecofriendly and affordable. Ultimately, PHAs will only become a real alternative to conventional plastics if both production and extraction are optimized.^5,11,12^

Although cost-effective, such lignocellulosic residual feedstocks require hydrolysis of their polysaccharides into the constituent monosaccharides (e.g., glucose, xylose) prior to their use for microbial cultivation.^13^ In addition, due to the recalcitrance of lignocellulosic biomass, a pretreatment stage is required before the enzymatic hydrolysis step. Pretreatment is typically accomplished by physicochemical processes, such as steam explosion, which may generate degradation products from sugars (furan derivatives and weak acids, e.g., furfural and formic acid) and lignin (phenolics), potentially toxic to PHA-producing microorganisms.^14^ The intrinsic variability in the operation of physical pretreatments (e.g., heating and cooling profiles on steam explosion), together with biomass compositional variability, often results in variability in the hydrolysates’ composition,^15,16^ which impacts PHA production performance.

Eucalyptus bark, particularly in Portugal, where eucalyptus species constitutes the major raw material used in pulp and paper mills, and its plantations cover approximately 812,000 ha,^17^ has recently been considered a promising feedstock for PHA production by several bacterial strains^18^ following its enzymatic conversion into a sugar-rich hydrolysate containing glucose and xylose. Rodrigues et al.^18^ reported the ability of *Burkholderia thailandensis* DSM 13276, a bacterium characterized by high metabolic versatility and the ability to simultaneously utilize mixed C5 and C6 sugars, to achieve a P­(3HB) content in the cells of 12 wt %. This outcome, although promising, was still low and requires further optimization to render the bioprocess cost-effective. Additionally, to further enhance the sustainability of P­(3HB) production, biopolymer recovery methods must also prioritize eco-friendliness. Conventional recovery methods, often reliant on toxic chlorinated solvents, raise environmental and safety concerns that limit the process’s scalability.^19^ Enzymatic digestion exhibits a great potential as an alternative to be applied due to its features as a 100% biological and highly selective process.^20,21^ Although scalability is currently limited by the cost of enzymatic cocktails, this route is essential to position PHAs as high-value functional materials rather than mere commodity plastic substitutes.^19,22^ Unlike sodium hypochlorite, which causes nonselective oxidation and severe chain scission, drastically reducing the polymer’s molecular weight, enzymatic lysis preserves its native structural integrity and mechanical performance. Furthermore, it avoids the environmental and safety risks associated with toxic solvents like chloroform.^5^ By maintaining the polymer’s premium properties, enzymatic recovery ensures that PHAs meet the rigorous standards for high-end applications, such as the biomedical sector,^23^ while fully aligning with the principles of a circular bioeconomy.^22^

Thus, the present study aimed at improving the previously reported P­(3HB) production by *B. thailandensis* DSM 13276 from eucalyptus bark enzymatic hydrolysate^18^ by adjusting the medium composition and integrating the bioprocess with biopolymer extraction through enzymatic digestion of the bacterial cells. The physical–chemical characteristics of P­(3HB) recovered from the bacterial cells were assessed to validate the proposed integrated production and extraction process.

## 2 Experimental Section

### 2.1 Feedstock Preparation

#### 2.1.1 Preparation of Eucalyptus Bark Hydrolysate

Hydrolysates were produced from *Eucalyptus globulus* bark supplied by the pulp mill of Cacia (Aveiro, Portugal) from The Navigator Company. The collected bark, consisting of the byproduct obtained from the debarking of wood rolls, was crushed in hammer or knife crushers in the mill, reducing its size and mitigating the fibrous effect that hinders the bark handling/transport. As previously reported,^14^ this bark was chemically characterized as-received by the authors, exhibiting a moisture content of 41 wt % and containing 49.9 g/100 g_o.d._ of total polysaccharides, of which 33.3 g/100 g_o.d._ and 12.2 g/100 g_o.d._ were glucan and xylan, respectively. Hydrolysates were obtained by applying enzymatic hydrolysis with a commercial cellulolytic enzymatic cocktail (Novonesis (Kongens Lyngby, Denmark) - Cellic CTec3 HS), as described by Rodrigues et al.^18^ Briefly, eucalyptus bark was processed by a proprietary noncatalyzed steam explosion technology, performed as a two-step pretreatment at 205 °C (17.5 bar) for 10 and 3 min, respectively. The resulting solid fraction (85% of the pretreated biomass on an oven-dried basis, after washing) was chemically characterized as previously described by the authors,^18^ demonstrating that the cellulose (glucan) was almost completely (97.3%) retained in the solid, whereas there was an extensive (58.2%) solubilization of the xylan. This pretreated bark was submitted to enzymatic hydrolysis with an enzyme load of 3 wt %, at an initial solid concentration (oven-dried basis) of 175 g/L, at 50 °C, for 48 h. The hydrolysate mostly comprised glucose (68.8 g/L) and xylose (8.47 g/L), corresponding to enzymatic hydrolysis yields of 92.3% and 61.8%, respectively, for conversion of cellulose and xylan to their constituent monosaccharides. It was immediately centrifuged (12,000*g*, 15 min) to eliminate unreacted solids and stored frozen. When thawed for use, aiming to reduce its protein content, the hydrolysate was further processed by ultrafiltration (UF) using a cross-flow system equipped with a tubular membrane module KLEANSEP (ALSYS, Salindres, France) K01, with a BX ceramic membrane (0.16-m2 membrane area) with 15 kDa molecular weight cutoff. UF was accomplished by applying a constant transmembrane pressure of 0.7 bar in the total recycling mode, providing a permeate flux of 24.4 L/(m^2^ h). The collected permeate was used (properly diluted) for culture medium preparation.

#### 2.1.2 Characterization of the Hydrolysate

The hydrolysates were evaluated in terms of color, pH, conductivity, and contents in sugars (glucose and xylose), ammonium, total protein, furfural, and 5-hydroxymethylfurfural (5-HMF), and organic acids (formic and acetic). Glucose and xylose concentrations were determined through high-performance liquid chromatography (HPLC), using a CarboPac PA10 column (Dionex, Sunnyvale, CA) coupled with an amperometric detector, as described by Pereira et al.^25^ Anhydrous D-(+)-glucose (99%) and D-(+)-xylose (99%) were used as standards at concentrations between 1 and 100 ppm. Ammonium concentration was determined by colorimetry in a segmented flow analyzer (Skalar 5100, Skalar Analytical). The total protein content was estimated using the total nitrogen kit (LCK338 Laton) and applying the Kjeldahl method by multiplying the result by the conventional factor 6.2 to obtain the crude protein content. Furfural, 5-HMF, formic acid, and acetic acid concentrations were determined by HPLC using an Aminex HPX-87H (Bio-Rad, Hercules, CA) equipped with a UV–vis detector, as described by Rodrigues et al.^18^ The detection was performed at 210 nm (for acetic and formic acids) and 280 nm (for furfural and 5-HMF). Standard solutions were prepared using glacial acetic acid (Fisher Chemical) and formic acid (Sigma-Aldrich) at concentrations ranging from 0.01 to 1 g/L, furfural (Sigma-Aldrich) at concentrations ranging from 0.0196 to 0.392 g/L, and 5-HMF (Sigma-Aldrich) at concentrations ranging from 0.00925 to 0.185 g/L.

### 2.2 Biopolymer Production

#### 2.2.1 Microorganism and Media

*Burkholderia thailandensis* DSM 13276 was used in all assays. Luria–Bertani (LB) broth (10 g/L NaCl, 10 g/L Bacto tryptone, 5 g/L yeast extract; pH 7.0) was used for preinoculum preparation, while Medium E*^18^ was used for inocula preparation and the bioreactor cultivation assays. Medium E* was supplemented with the eucalyptus bark enzymatic hydrolysate to give a sugar concentration of approximately 30 g/L. The media pH value was set to 7.0 by adding NaOH before autoclaving at 121 °C and 1 bar for 20 min.

#### 2.2.2 Inoculum Preparation

The culture was plated onto CHROMagar Orientation plates and incubated for 48 h at 30 °C. A single colony, isolated from the agar plate, was inoculated in LB broth (20 mL) in 100 mL Erlenmeyer flasks and incubated for 24 h at 30 °C in an orbital shaker (200 rpm). This preculture (20 mL) was transferred into a 500 mL baffled shake flask with 200 mL of Medium E* prepared as described above and incubated under the same conditions for 72 h to obtain the inoculum for the bioreactor cultivation experiments.

#### 2.2.3 Bioreactor Cultivation

*B. thailandensis* DSM 13276 was cultured in a 2 L bioreactor (BioStat B-Plus, Sartorius, Germany) with 10% (v/v) inoculum (200 mL) operated in batch mode. The temperature was maintained at 30 ± 0.1 °C, and the pH was controlled at 7.0 ± 0.1 by the automatic addition of 2 M HCl or 5 M NaOH. A constant airflow rate of 2 standard liters per minute was maintained throughout the cultivation run, and the dissolved oxygen (DO) concentration was set at 30% of the air saturation by automatically adjusting the stirrer speed between 200 and 2000 rpm. Antifoam A (Sigma-Aldrich) was automatically added to prevent foam formation.

Samples (12 mL) were collected and centrifuged (9000*g*, 15 min) for cell separation. The supernatant was used for quantification of sugars, ammonium, furfural, 5-HMF, acetic acid, and formic acid, while the pellets were used for cell dry weight (CDW) and P­(3HB) quantification. The cell pellets were washed with deionized water and freeze-dried, and the CDW was gravimetrically determined by weighing the dry pellets. The P­(3HB) content in the biomass and the biopolymer composition were determined by gas chromatography (GC), following the methanolysis method described by Rodrigues et al.^18^ Sugars, ammonium, protein, furfural, 5-HMF, formic acid, and acetic acid were quantified as described above.

#### 2.2.4 Calculations

The maximum specific cell growth rate (μ_max_, h^–1^) was calculated using the slope of the exponential phase of Ln *X_t_* versus time, where *X_t_* g/L represents the cells without P­(3HB) (rest biomass), at time *t* (h). The rest biomass was calculated as follows

1 $$X_{t}={\mathrm{CDW}}_{t}-{P\left(3\mathrm{HB}\right)}_{t}$$

where CDW*_t_* (g/L) is the cell dry weight, and P­(3HB) (g/L) is the polymer concentration at time *t* (h). The P­(3HB) concentration was determined based on the polymer content within the bacterial cells, as a percentage of the cells’ dry weight (wt %). The polymer and growth yields on a substrate basis (*Y*_P/S_, g_p_/g_s_) were determined as follows

2 $$Y_{P/S}=\Delta P/\Delta S$$

3 $$Y_{X/S}=\Delta X/\Delta S$$

where Δ*P* (g/L) is the synthesized P­(3HB), Δ*X* (g/L) is the rest biomass, and Δ*S* (g/L) is the total sugars consumed over the assays. The volumetric productivity (*r*_p_, g/(L day)) was calculated by dividing the produced P­(3HB) (*P*, g/L) by the cultivation time (Δ*t*, day).

### 2.3 P­(3HB) Extraction and Purification

#### 2.3.1 Standard Solvent Extraction

The cell pellet obtained by centrifuging the culture broth (9000*g*, for 20 min) was washed with deionized water and freeze-dried. The P­(3HB) was extracted from the cells through Soxhlet extraction with chloroform (Sigma-Aldrich, 99–99.4%) and purified by precipitation in ice-cold ethanol (LabChem, 96%), as described by Rebocho et al.^24^

#### 2.3.2 Enzymatic Extraction

For the enzymatic extraction, the method described by Martino et al.^23^ was used, with minor modifications. The culture broth (1 L) was centrifuged (9000*g*, for 20 min), and the pellet was weighed. Then, 1 L of a Na_2_HPO_4_ buffer solution (pH 8.3) was mixed with the wet cell pellet to give 20% (w/v) content. Alcalase (0.3 AU g/g, where AU = Anson Units), sodium dodecyl sulfate (SDS, 0.3 g/g), and ethylenediaminetetraacetic acid (EDTA, 0.01 g/g) were added to the suspension, which was incubated at 55 °C for 1 h under constant stirring (200 rpm). The resulting P­(3HB) granules were collected by centrifugation (9000*g*, for 20 min), washed twice with deionized water (2× 1 L), and dried at 70 °C, in a ventilated oven, until constant weight was reached (∼48 h). All chemicals were obtained from Sigma-Aldrich and used directly without any additional purification steps.

### 2.4 Polymer Characterization

#### 2.4.1 Fourier Transform Infrared Spectroscopy

Fourier transform infrared spectroscopy (FTIR) was performed with a PerkinElmer Spectrum, two spectrometers at room temperature, with 10 scans between 400 and 4000 cm^–1^ resolution.

#### 2.4.2 Molecular Mass Distribution

The samples were dissolved in chloroform (1 g/L) at 70 °C for 1 h. The solutions were filtered (0.22 μm PTFE, Labfil) and analyzed by a size exclusion chromatography (SEC) system (Waters Millennium), as described by Rebocho et al.^24^ Monodisperse polystyrene standards (Sigma-Aldrich), with molecular weights (*M*_w_) ranging from 800 Da to 504 kDa, were used. The relative *M*_w_ was calculated according to the universal calibration method using Waters Millennium SEC software.

#### 2.4.3 Thermal Properties

Differential scanning calorimetry (DSC) was performed using a differential scanning calorimeter Discovery Series DSC25 (TA Instruments, New Castle, DE) coupled to a cooling system 90 (TA Instruments Refrigerated cooling system 90), as described by Rebocho et al.^25^ The melting temperature (*T*_m_, °C) was taken at the endothermic peak’s minimum. Thermogravimetric analysis (TGA) was done with a thermogravimetric equipment Labsys EVO (Setaram, France), as described by Esmail et al.,^26^ in a temperature range from 25 to 800 °C. The degradation temperature (*T*_deg,5%_) was defined as the temperature at which the sample showed a 5% mass loss, indicating the onset of significant thermal degradation. The maximum degradation temperature (*T*_deg,max_) was determined as the temperature corresponding to the peak’s maximum degradation rate during the main mass loss.

#### 2.4.4 X-ray Diffraction

X-ray diffraction (XRD) was performed with an X-ray diffractometer (PANalytical X′Pert PRO MRD), with a monochromatic Cu Kα radiation source (45 kV and 40 mA), to scan the samples over a 2θ range from 10 to 90° using a 10°/min scan rate (continuous scanning mode). The resulting diffractograms were used to identify the crystalline and amorphous phases of the polymer and to calculate the degree of crystallinity (χ_c_).

## 3 Results and Discussion

### 3.1 Hydrolysate Characterization

The sugar solution obtained by enzymatic hydrolysis of the pretreated eucalyptus bark contained glucose (68.6 g/L) and xylose (8.47 g/L) together with a high protein titer (184.6 g/L), corresponding to the enzyme cocktail content (exhibiting cellulolytic and hemicellulolytic activities). Thereby, aiming to recover the enzyme and reduce the protein content (rendering it more suitable for bacterial cultivation), the hydrolysate was further processed by ultrafiltration through a 15 kDa molecular weight cutoff membrane. As expected, the collected permeate, exhibiting a clear amber-brown coloration with no significant turbidity, contained a much lower protein titer of 35.5 ± 5.23 g/L together with 54.9 ± 1.21 g/L of glucose and 7.3 ± 0.03 g/L of xylose (Table 1). Thereby, the membrane used accomplished 87.5% retention of protein, with extensive glucose and xylose permeations, corresponding, respectively, to 70.6 and 78.9% transmissions. The permeate also exhibited low concentrations of formic acid (0.08 ± 0.06 g/L), acetic acid (0.48 ± 0.05 g/L), and ammonium (0.32 ± 0.05 g/L), while furfural and 5-HMF were not detected (Table 1).

**Table 1:** Physical and Chemical Characterizations of the Eucalyptus Bark Ezymatic Hydrolysate Obtained after Ultrafiltration and Used for Bacterial Cultivation^a^

parameter	hydrolysate
total protein (g/L)	35.50 ± 5.23
ammonium (g/L)	0.32 ± 0.05
glucose (g/L)	54.9 ± 1.21
xylose (g/L)	7.3 ± 0.03
furfural (mg/L)	n.d.
5-HMF (mg/L)	n.d.
formic acid (g/L)	0.08 ± 0.06
acetic acid (g/L)	0.48 ± 0.05

a n.d., not detected.

Acetic acid, primarily originating from the deacetylation of hemicellulose acetyl groups, and formic acid, formed through carbohydrate breakdown under acidic or high-temperature conditions,^27^ are among the most significant microbial inhibitors in lignocellulosic hydrolysates,^28^ but the low values found in the eucalyptus bark hydrolysate obtained after ultrafiltration were not expected to impair bacterial activity. 5-HMF and furfural, which are commonly found in lignocellulosic hydrolysates, resulting from hexoses (e.g., glucose) and pentoses (e.g., xylose) degradation,^29^ are usually associated with inhibition of microbial activity as they disrupt key metabolic pathways, leading to decreased cultivation efficiency.^30^ Probably due to the hydrolysate’s processing, neither compound was detected, rendering it less prone to cause inhibition of bacterial activity. The detected ammonium content, likely originating from the breakdown of glycoproteins present in the eucalyptus bark^31^ or from the degradation of the enzymes used for bark hydrolysis, correlates with the hydrolysate’s protein content. Both ammonium and protein are nitrogenous compounds that can be used as nitrogen sources for microbial cultivation.^28^

### 3.2 Biopolymer Production

The eucalyptus bark hydrolysate obtained by ultrafiltration was diluted in the cultivation medium to provide a sugar concentration of approximately 30 g/L. As shown in Figure 1, it provided an adequate source of nutrients for the production of P­(3HB) by *B. thailandensis*. The culture exhibited no lag phase (Figure 1), displaying a maximum specific cell growth rate of 0.11 h^–1^ (Table 2) and reaching a CDW of 4.30 ± 0.12 g/L after around 25 h of cultivation, upon ammonia depletion. The displayed cell growth rate is near that reported by Rodrigues et al.^18^ (0.13 h^–1^), but a higher CDW was reached at the end of the experiment (7.67 ± 0.16 g/L) (Table 2). Moreover, a considerably higher biopolymer content in the cells (60.0 ± 2.19 wt %) was obtained compared to that reached by Rodrigues et al.^18^ (12.0 wt %), which aligns with the range of values reported for *B. thailandensis* and other *Burkholderia* species (46–64 wt %) (Table 2). P­(3HB) production (4.60 ± 0.17 g/L) and the overall volumetric productivity (1.36 g/(L day)) were also higher than those reported by Rodrigues et al.^18^ (1.10 g/L and 0.55 g/(L day), respectively) (Table 2). More importantly, P­(3HB) synthesis was clearly favored over cell growth, as shown by the higher polymer yield (0.19 g_P(3HB)_/g_sugar_), compared to the growth yield (0.12 g_X_/g_sugar_).

**Figure 1:**
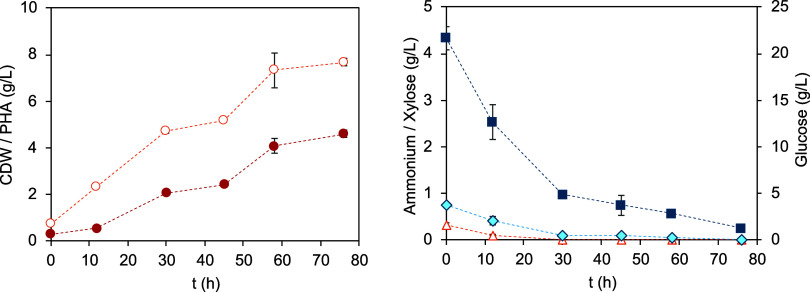
Cultivation profile of *B. thailandensis* in the enzymatic eucalyptus bark hydrolysate (red ring open, CDW; red circle solid, P­(3HB); blue box solid, glucose; blue tilted square open, xylose; red triangle up open, ammonium).

**Table 2:** Kinetic and Stoichiometric Parameters for Cultivation of Several *Burkholderia* spp. in Different Feedstocks^a^

bacterium	feedstock	cultivation mode	μ_max_ (h^–1^)	CDW (g/L)	P(3HB) (wt %)	P(3HB) (g/L)	*r*_p_ (g/(L day))	*Y*_x/s_ (g_X_/g_sugar_)	*Y*_p/s_ (g_P(3HB)_/g_sugar_)	references
*B. thailandensis* DSM 13276 (E264)	Eucalyptus bark hydrolysate	Batch bioreactor	0.11	7.67 ± 0.16	60.0 ± 2.19	4.60 ± 0.17	1.36	0.12	0.19	this study
	Eucalyptus bark hydrolysate	Batch bioreactor	0.13	6.32	12.0	1.10	0.55	0.13	0.02	^18^
	NB medium supplemented with 4 wt % used cooking oil	Batch bioreactor	0.03	12.6 ± 0.8	60.0 ± 0.7	7.5 ± 0.4	1.5 ± 0.1	n.a.	n.a	^33^
	Glucose/xylose	Baffled shake flasks	n.a.	5.99 ± 0.21	64.0 ± 4.9	3.84 ± 0.39	0.48	n.a.	0.19 ± 0.03	^34^
*B. sacchari* IPT101	Bagasse Hydrolysate	Batch, bioreactor	0.24	4.4	62.0	2.73	n.a.	n.a.	0.39	^35^
*B. sacchari* LMG19450	Xylose	Fed-Batch	0.19	13.10 ± 0.57	61.70 ± 5.23	n a	0.12 ± 0.00	0.24	0.37	^36^
*B. cepacia* ATCC 17759	Wood Hydrolysate	Fed-batch, bioreactor	n.a.	16.97	51.4	8.72	n.a.	n.a.	0.19	^37^
*B. glumae* MA13	Glycerol	Baffled shake flasks	n.a.	4.20	46.27	1.94	n.a.	n.a.	0.21	^38^

a n.a., data not available; μ_max_, maximum specific cell growth rate; *r*_p_, volumetric productivity; *Y*_x/s_, growth yield; *Y*_p/s_, product yield; X, active biomass.

The protein content present in the cultivation medium (35.5 ± 5.23 g/L) remained unchanged during the assay, while the acetic (4.5 ± 0.7 mg/L) and formic (18.9 ± 2 mg/L) acids were consumed early in the cultivation run, which likely contributed to support bacterial cell growth. The low concentrations of acetic and formic acids seem to have had no significant effect on the cell growth or P­(3HB) production. This aligns with the findings of Marudkla et al.^32^ who reported that acetic acid concentrations up to 0.5 g/L had no negative impact on cell growth or P­(3HB) production by *Cupriavidus necator* DSM 545. These results suggest that the concentrations of organic acids in this study were below the threshold that affects microbial performance.

Contrasting with the study of Rodrigues et al.,^18^ in which higher glucose (68.6 ± 0.7 g/L) and xylose (8.47 ± 0.17 g/L) concentrations apparently favored cell growth and hindered P­(3HB) synthesis, lower levels of inhibitors and a more balanced nutrients’ composition (carbon and nitrogen) have favored biopolymer accumulation by *B. thailandensis*. The presence of higher levels of formic acid (0.494 ± 0.021 g/L) and acetic acid (1.17 ± 0.038 g/L) together with vestigial concentrations of 5-HMF and furfural in the undiluted hydrolysate in Rodrigues et al.^18^ study seems to have diverted the cells’ metabolic activity from P­(3HB) production, suggesting that the cells likely redirected energy toward detoxification rather than biopolymer synthesis. This shift in metabolic resources is supported by previous studies indicating that under high-stress conditions, cells prioritize survival mechanisms over polymer production,^39^ as furfural and 5-HMF are known to interfere with key enzymes in glycolysis and the TCA cycle, such as alcohol dehydrogenase and pyruvate dehydrogenase, which are essential for energy production and efficient P­(3HB) synthesis.^30^ These results underscore the critical role of medium composition in maximizing microbial performance, wherein reducing the levels of inhibitors impacts cell growth and P­(3HB) synthesis.

### 3.3 Biopolymer Extraction

The biopolymer produced by *B. thailandensis* was recovered from the bacterial biomass using the enzyme alcalase for cell disruption, followed by nonpolymer cell mass (NPCM) dissolution in the aqueous extraction medium and biopolymer purification by washing with water. For comparison, solvent extraction with chloroform and purification by precipitation in cold ethanol were also performed. As shown in Figure 2, both methods yielded white, high-purity P­(3HB), but the enzymatic extraction resulted in a fine powder (Figure 2-A), while the solvent method resulted in aggregated clumps (Figure 2-B), occurring due to the rapid precipitation of P­(3HB) when dissolved in chloroform and precipitated in ethanol.

**Figure 2:**
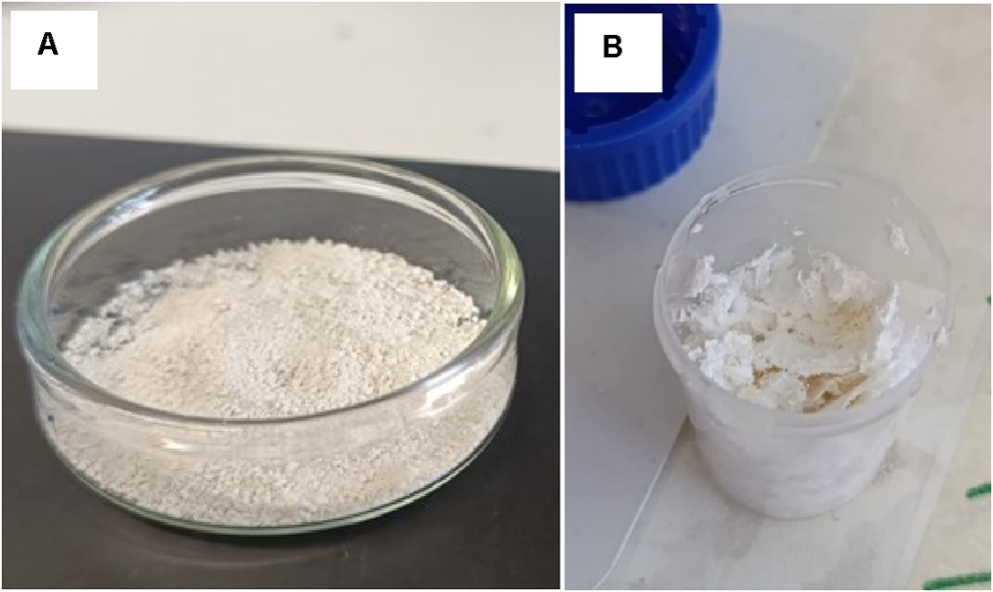
P­(3HB) extracted from the biomass produced by *B. thailandensis* using the hydrolysate as feedstock by applying the enzymatic method (A) and by applying solvent extraction with chloroform (B).

An extraction efficiency of 96% P­(3HB) was achieved with the enzymatic method, slightly lower than the 98% efficiency obtained with chloroform. Notably, the enzymatically extracted P­(3HB) exhibited exceptional purity (100 ± 3.38%), consistent with the purity obtained using the chloroform method (99 ± 4.12%). This purity aligns with that reported by Martino et al.^23^ who achieved ∼ 94% purity of amorphous P­(3HB) granules from *C. necator* using the same procedure. It is substantially higher than the 83.1% recovery (with lower implied purity) obtained by Neves et al.^11^ using alcalase 2.4 L (with no additional surfactants), under optimized thermal pretreatment (121 °C, 15 min), on *C. necator*.^11^ for P­(3HB) extraction, contrasting with the present protocol—combining mild heat, optimized pH, and enzyme loading—that reached 96% yield and 100% purity.

The overall yield achieved in this bench-scale study for the conversion of eucalyptus bark into biopolymers is already very satisfactory. Indeed, when considering the whole process integrating all of the stages, i.e., from pretreatment (by steam explosion) to the enzymatic polymer extraction, the proposed process provides 77.1 kg of pure P­(3HB) per metric ton (oven-dried basis) of eucalyptus bark as collected in the pulp mill. Ultrafiltration of the hydrolysate prior to bacterial cultivation, hereby introduced, has given rise to a higher P­(3HB) yield compared to that reported by Rodrigues et al.^18^ Moreover, it can bring cost savings by allowing reusing the enzymes recovered in the UF’s retentate.

In terms of scalability, the transition to an integrated enzymatic bioprocess is supported by the use of safer, aqueous-based conditions that align with standard industrial infrastructure. The extraction’s scalability is particularly enhanced by the elimination of chlorinated solvents, reducing environmental risks at high volumes. When integrated, these enzymatic steps create a robust framework for industrial-scale biorefineries, provided that enzyme reuse strategies are implemented to ensure economic competitiveness.

### 3.4 P­(3HB) Characterization

FTIR spectroscopy analysis of the P­(3HB) produced by *B. thailandensis* using the eucalyptus bark hydrolysate, recovered from the biomass by solvent extraction with chloroform, displayed the characteristic peaks (Figure 3) consistent with literature-reported spectra for this homopolymer, thus confirming that the feedstocks’ composition had no significant impact upon the biopolymer’s chemical structure. Moreover, the sample extracted by enzymatic treatment with alcalase was characterized by an identical spectrum, with only minor differences in some peaks’ intensity (Figure 3), thus showing that the use of an aqueous-based extraction procedure had no significant impact on the biopolymer’s chemical structure.

**Figure 3:**
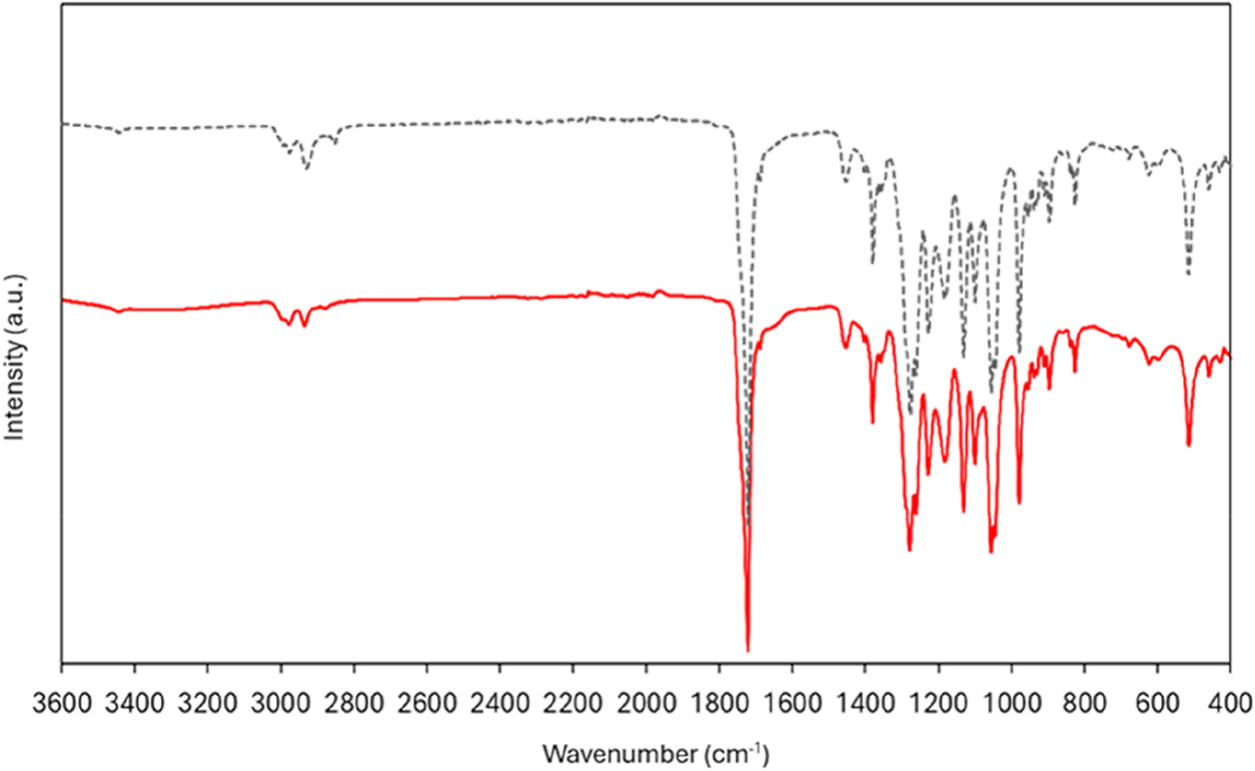
Fourier transform infrared spectroscopy (FTIR) spectra of the P­(3HB) produced by *B. thailandensis* using the eucalyptus bark hydrolysate, recovered by chloroform extraction (dashed line) and by enzymatic treatment (full line).

The peaks observed at around 2934 and 2979 cm^–1^, observed for all samples (Figure 3), correspond to the C–H stretching vibrations of methylene groups, which are typically reported for P­(3HB) in the range of 2933–2975 cm^–1^.^49^ The prominent peak at 1721 cm^–1^ can be assigned to the C=O stretching of the ester carbonyl group associated with the crystallinity of P­(3HB), commonly reported to be found at around 1724 cm^–1^.^40,41^ Additionally, the C–O stretching bands observed between 1054 and 1280 cm^–1^ reflect the crystallinity of the polymer structure.^41^ These results confirm that the P­(3HB) produced in each batch reactor closely matches the expected FTIR profile of the P­(3HB) homopolymer, with only minor shifts in peak positions that remain within typical variability ranges.^42^

As shown in Table 3, all P­(3HB) samples presented similar molecular mass distributions, irrespective of the extraction method used for their recovery from the bacterial cells. In fact, the P­(3HB) enzymatically extracted displayed a Mw (3.68 × 10^5^ Da) identical to that of the chloroform-extracted sample (3.86 × 10^5^ Da), with similar PDI (1.86 and 1.78, respectively), as showed by Martino et al.,^23^ who reported no detectable polymer degradation after alcalase/SDS/EDTA treatment, and contrasts with potential chain scission seen in harsher chemical routes.^43^ Overall, the Mw of the samples is within the range reported for P­(3HB) produced by *B. thailandensis* (2.16 × 10^5^–1.381 × 10^6^ Da),^34^ as well as by other bacteria (10^4^ – 10^6^ Da),^44^ including the commercial product P­(3HB)-Aldrich P­(3HB), from Sigma-Aldrich Co. (5.06 × 10^5^ ± 900 Da).

**Table 3:** Average Molecular Weight (*M*_w_), Polydispersity Index (PDI), Crystallinity Index, Crystallite Size, Melting Temperature (*T*_m_), Thermal Degradation Temperature at 5% Mass Loss (*T*_deg,5%_), and Maximum Degradation Temperature (*T*_deg,max_) of the P­(3HB) Produced by *B. thailandensis* Grown in Eucalyptus Bark Enzymatic Hydrolysate and Recovered from the Biomass by Enzymatic Treatment with Alcalase or by Chloroform Extraction

P(3HB) extraction procedure	solvent extraction with chloroform and purification by ethanol precipitation	aqueous-based enzymatic extraction
*M*_w_ (×10^5^ Da)	3.86	3.68
PDI	1.78	1.86
crystallinity index (%)	90	81.6
crystallite size (nm)	7.77	10.35
*T*_m_ (°C)	175	170
*T*_deg,5%_ (°C)	277	255
*T*_deg,max_ (°C)	294	280
char yield_800 °C_ (%)	0	2.5

The crystallinity index of the P­(3HB) subjected to chloroform extraction (90%) (Table 3) is slightly higher than those reported in other studies^45,46^ for P­(3HB) (50–80%) but closely matches the values reported by Zainuddin et al.^47^ (92%), suggesting that the crystalline structure of the biopolymer was well-preserved. Interestingly, the enzymatically extracted P­(3HB) exhibited a lower crystallinity index (81.6%) (Table 3) compared with the P­(3HB) extracted with chloroform (≥90%). This difference could be attributed to the aqueous environment involved in the enzymatic extraction procedure and the milder conditions. Water may act as a plasticizer, inhibiting crystallization by increasing polymer chain mobility.^48^ In terms of crystallite size, slight differences were noticed between the P­(3HB) recovered from the biomass by chloroform extraction (7.77 nm) (Table 3) (Figure S1) and by the enzymatic procedure (10.35 nm), which is comparable to the reference value (10.74 nm),^47^ indicating that the samples retained the average crystallite size associated with P­(3HB)’s structural integrity.

Slightly lower melting temperatures (170 °C) were observed for the enzymatically extracted P­(3HB), compared to the chloroform-extracted sample (175 °C) (Table 3), which are all consistent with the reported range for P­(3HB) (170–190 °C).^25,46,49,50^ The same trend was observed for the degradation temperature, with that of P­(3HB) extracted with alcalase displaying a slightly lower value (280 °C) than that extracted with chloroform (294 °C).

Figure 4 displays the TGA profiles of the chloroform-extracted P­(3HB) sample synthesized from the hydrolysates by applying the two tested procedures for extraction. The absence of mass loss up to 160 °C in the spectrum of the chloroform-extracted samples confirms the effective removal of residual solvent used during polymer extraction, as previously described by Pradhan et al.^50^ The P­(3HB) extracted through the enzymatic method showed a 1.77% mass decrease between 30 and 140 °C, probably derived from the loss of adsorbed water.^51^ There was no significant detectable weight loss in any of the P­(3HB) samples up to around 230 °C, indicating a high thermal stability. However, the enzymatically extracted P­(3HB) exhibited a 5% mass loss at 255 °C, while the chloroform-extracted sample displayed the same degree of mass loss at a higher temperature (277 °C) (Table 3), consistent with their higher thermal stability. All samples exhibited a single-stage degradation process with weight losses in the range of 93–97% (Figure 4). The samples showed an average char yield of 1.89 ± 2.40%, concomitant with their purity degree.

**Figure 4:**
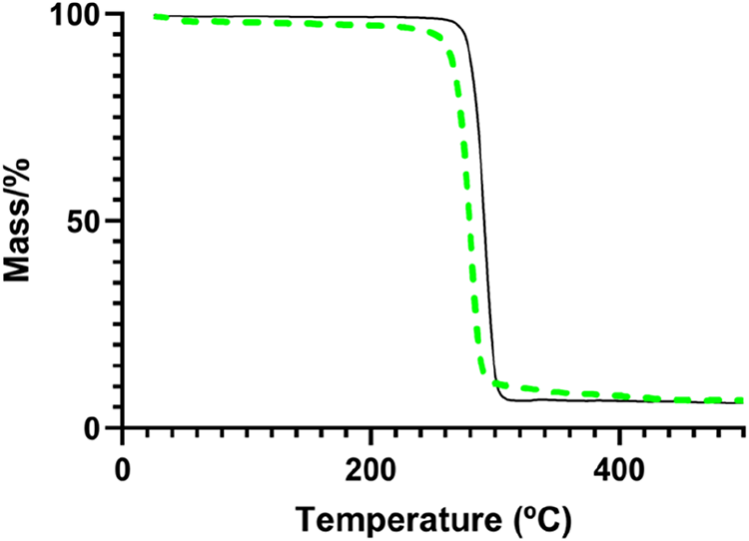
Thermogravimetric analysis (TGA) curves of the P­(3HB) samples produced by *B. thailandensis* from the enzymatic eucalyptus bark hydrolysate, recovered by chloroform extraction (black, full line) and with alcalase treatment (green, dashed line).

Despite the slight differences observed for the P­(3HB) samples produced by *B. thailandensis* from the eucalyptus bark enzymatic hydrolysate, recovered by either extraction procedure, their thermal behavior aligns with that reported for P­(3HB) samples. For example, Blunt et al.^34^ observed degradation temperatures ranging from 278 to 300 °C for P­(3HB) produced by *B. thailandensis* E264 using different substrates (e.g., glucose, xylose, glycerol). Similarly, Rebocho et al.^25^ reported a *T*_deg_ of 290 °C and a weight loss (Δ*m*) of 97% for the P­(3HB) produced by *C. necator.* These ranges are also consistent with the findings of Kervran et al.^52^ which have demonstrated that P­(3HB) degrades in a single step within the temperature range of 220–290 °C.

## 4 Conclusions

This study demonstrated the feasibility of applying enzymatic technologies for the upstream (enzymatic saccharification of eucalyptus bark) and downstream (aqueous-based enzymatic procedure for biopolymer extraction) of the bioprocess for P­(3HB) production by *B. thailandensis* using a residual lignocellulosic biomass as the sole raw material. The ultrafiltration step introduced in the upstream feedstock processing, besides increasing P­(3HB) production compared to previous studies, also allows for partial enzyme recovery and reuse, which greatly contributes to rendering the overall process more cost-effective. The use of alcalase for P­(3HB) extraction represents a valuable alternative to the use of hazardous chlorinated solvents, resulting in a biopolymer characterized by physical and chemical properties identical to the chloroform-extracted samples. Even though the costs associated with the enzyme add to the overall process costs, those can be significantly reduced by enzyme recovery and reuse. Overall, the proposed upstream and downstream enzymatic procedures boost the environmental sustainability of the P­(3HB) production process, fully aligned with the circular bioeconomy principles.

## Supplementary Material



## Data Availability

Data and materials will be provided on request.

## Notes

**Figure S1**. Peak positions and intensity (left), alignment of produced P­(3HB) positions with standard P­(3HB) diffraction data from the PDF-2 database, provided by the International Centre for Diffraction Data (ICDD) (right), for the P­(3HB) produced by *B. thailandensis* from the eucalyptus hydrolysate (PDF)
