# Supplementary material for: Sustainable Production of Poly(3-hydroxybutyrate) Using Eucalyptus Bark: Integration with Green Downstream Processing
Source: ACS Sustain Chem Eng. 2026 Feb 9;14(7):3749–57. doi: 10.1021/acssuschemeng.5c14243 (PMC12934527; doi:10.1021/acssuschemeng.5c14243)
Supplement: Supplementary file 1 [file sc5c14243_si_001.pdf]

Supporting Information

Sustainable Production of Poly(3-hydroxybutyrate) using Eucalyptus Bark: Integration with Green Downstream Processing

João Matias [1,2,§], Thomas Rodrigues [1,2,§], Cristiana A. V. Torres [1,2], Susana Marques [3], Belina Ribeiro [3], Francisco Gírio [3], Maria A. M. Reis [1,2], Filomena Freitas [1,2,]*

[1] Associate Laboratory i4HB, Institute for Health and Bioeconomy, School of Science and Technology, NOVA University Lisbon, 2829-516 Caparica, Portugal; jp.matias@campus.fct.unl.pt (J.M.); ta.rodrigues@campus.fct.unl.pt (T.R.); c.torres@fct.unl.pt (C.A.V.T.); amr@fct.unl.pt (M.A.M.R.); a4406@fct.unl.pt (F.F.)

[2] UCIBIO—Applied Molecular Biosciences Unit, Department of Chemistry, School of Science and Technology, NOVA University Lisbon, 2829-516 Caparica, Portugal

[3] Unidade de Bioenergia e Biorrefinarias, Laboratório Nacional de Energia e Geologia I.P., 2610-999 Lisboa, Portugal; susana.marques@lneg.pt (S.M.); belina.ribeiro@lneg.pt (B.R.); francisco.girio@lneg.pt (F.G.)

§ these authors contributed equally to this work

* Corresponding author: a4406@fct.unl.pt

Number of pages: 33

Number of figures: 4

Number of tables: 3

Number of equations: 3

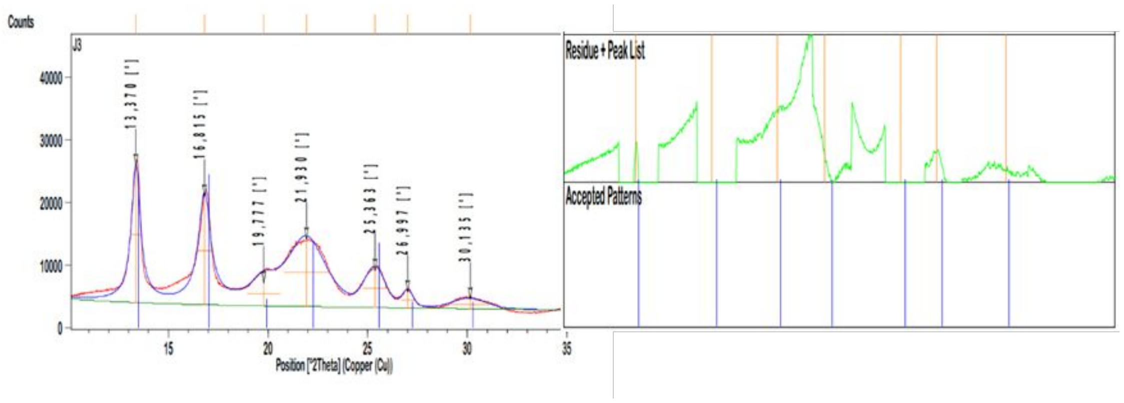

Figure S1 - Peak positions and intensity (left), alignment of produced PHB positions with standard PHB diffraction data from the PDF-2 database, provided by the International Centre for Diffraction Data (ICDD) (right), for the PHB produced by *B. thailandensis* from the eucalyptus hydrolysate.
